# Domestic waste disposal practice and perceptions of private sector waste management in urban Accra

**DOI:** 10.1186/1471-2458-14-697

**Published:** 2014-07-08

**Authors:** Ramatta Massa Yoada, Dennis Chirawurah, Philip Baba Adongo

**Affiliations:** 1 Department of Social and Behavioural Science, School of Public Health, University of Ghana, Post Office Box LG 13, Legon, Ghana; 2 Department of Community Health and Family Medicine, School of Medicine and Health Science, University for Development Studies, Post Office Box 1883, Tamale, Ghana

**Keywords:** Domestic waste, Solid waste disposal, Perceptions about waste, Ghana

## Abstract

**Background:**

Waste poses a threat to public health and the environment if it is not stored, collected, and disposed of properly. The perception of waste as an unwanted material with no intrinsic value has dominated attitudes towards disposal. This study investigates the domestic waste practices, waste disposal, and perceptions about waste and health in an urban community.

**Methods:**

The study utilised a mixed-method approach. A cross-sectional survey questionnaire and in-depth interview were used to collect data. A total of 364 household heads were interviewed in the survey and six key informants were interviewed with the in-depth interviews.

**Results:**

The results of the study revealed that 93.1% of households disposed of food debris as waste and 77.8% disposed of plastic materials as waste. The study also showed that 61.0% of the households disposed of their waste at community bins or had waste picked up at their homes by private contractors. The remaining 39.0% disposed of their waste in gutters, streets, holes and nearby bushes. Of those who paid for the services of private contractors, 62.9% were not satisfied with the services because of their cost and irregular collection. About 83% of the respondents were aware that improper waste management contributes to disease causation; most of the respondents thought that improper waste management could lead to malaria and diarrhoea. There was a general perception that children should be responsible for transporting waste from the households to dumping sites.

**Conclusion:**

Proper education of the public, the provision of more communal trash bins, and the collection of waste by private contractors could help prevent exposing the public in municipalities to diseases.

## Background

Globally, millions of tons of municipal solid waste are generated every day. Urban waste management is drawing increasing attention, as it can easily be observed that too much garbage is lying uncollected in the streets, causing inconvenience, environmental pollution, and posing a public health risk [1,2].

The problem of solid, liquid, and toxic-waste management in Africa has come with urbanization in the developing world. An important feature of the urbanization of the developing world is the rapid growth of cities and metropolitan areas. The high rate of urbanization in African countries implies a rapid accumulation of refuse. Social and economic changes that most African countries have witnessed since the 1960s have also contributed to an increase in the waste generated per capita [3,4]. As a result, municipal waste management constitutes one of the most crucial health and environmental issues facing managers of African cities [5,6]. Proper waste management is a public benefit and obligation. Improper waste disposal by one individual affects the entire citizenry, so, as a policy, countries have tasked every individual, establishment or institution to contribute significantly to the process of keeping their communities and environment clean [7-9].

In the colonial days, the population of the Ghana, then the Gold Coast, was below six million and waste was better managed. The waste generated in the 1920s was less voluminous and less complex than today, consisting largely of leaves, paper and wood products, with little plastic or hazardous chemicals [9]. The poor waste management situation in recent years has led to a high incidence of sanitation related illness, such as cholera, intestinal worms and typhoid. These are among the top ten diseases that have been recorded, which raises the alarm of a public health crisis [10-13]. In Ghana, problems are encountered at all levels of waste management, particularly, collection, transportation and disposal. Generally, existing public facilities, including sanitary facilities, are inadequate to serve the user population, and the sheer volume of municipal solid waste generated in the country’s urban centres is overwhelming. While existing waste disposal facilities are inadequate to deal with the quality and quantity of waste generated, more sophisticated systems are expensive and their maintenance requirements are high [14].

In Ghana, a study conducted at Kodiabe, which involved direct observations at disposal sites from five divisions, focused on the way in which refuse materials were disposed [15]. Another study conducted in Nigeria showed that the perception of domestic waste disposal indicates that people’s attitudes about and perceptions of sanitation issues contribute to the waste management problem [16]. Similarly, a study done in Khulna, Bangladesh found that city dwellers think because they pay taxes it is the sole responsibility of the city authority to provide them with a nuisance-free habitable city [17]. Typically, local governments are responsible for the collection and disposal of the wastes generated within their jurisdiction, as well as for the operation and maintenance of their equipment. However, local governments usually lack the authority and resources to provide a satisfactory and economically viable service. Effective and efficient solid waste management depends upon an equitable distribution of responsibilities, authority, and revenue between the national government and all the local governments [18]. General waste management in Ghana is perceived to be the responsibility of the Ministry of Local Government and Rural Development, which supervises the decentralized Metropolitan, Municipal and District Assemblies (MMDAs). However, regulatory authority is vested in the Environmental Protection Agency (EPA) under the auspices of the Ministry of Environment and Science. The MMDAs are responsible for the collection and final disposal of solid waste through their Waste Management Departments and their Environmental Health and Sanitation Departments. However, there is a growing perception that inadequate education about the importance of proper sanitation account for poor waste management practices in Ghana. Other factors accounting for this situation are poor attitudes and lack of concern about environmental issues, high levels of poverty and misguided waste disposal practices [19,20].

As in many developing countries, waste management in Ghana is a complex issue that has been a major issue on the priority list of successive governments, local authorities, and international donors in recent years. Waste management is a growing problem in Ghana, and despite large investments that have been made to meet the challenges of effective waste management in urban Ghana, there is little evidence that such efforts are having their expected effect [21]. Although huge capital investment is required to improve waste management, social and behavioural factors are also important if waste management in urban areas is to be successful. It is in this light that the current study aims to investigate community practices and perceptions about solid waste management and it implications for health in urban Accra.

## Methods

### Ethics statement

Ethical approval for this study was received from the Ethics Review Committee of the Ghana Health Service. The purpose of the study was explained to all participants, after which written and verbal consent was received from each participant. All participants were assured of anonymity and the confidentially of the information received from them. Permission also was received from the director of the Municipal Health Management Team (MHMT) and the District Assembly that is responsible for waste management in the municipality.

### Study area

**Figure 1:**
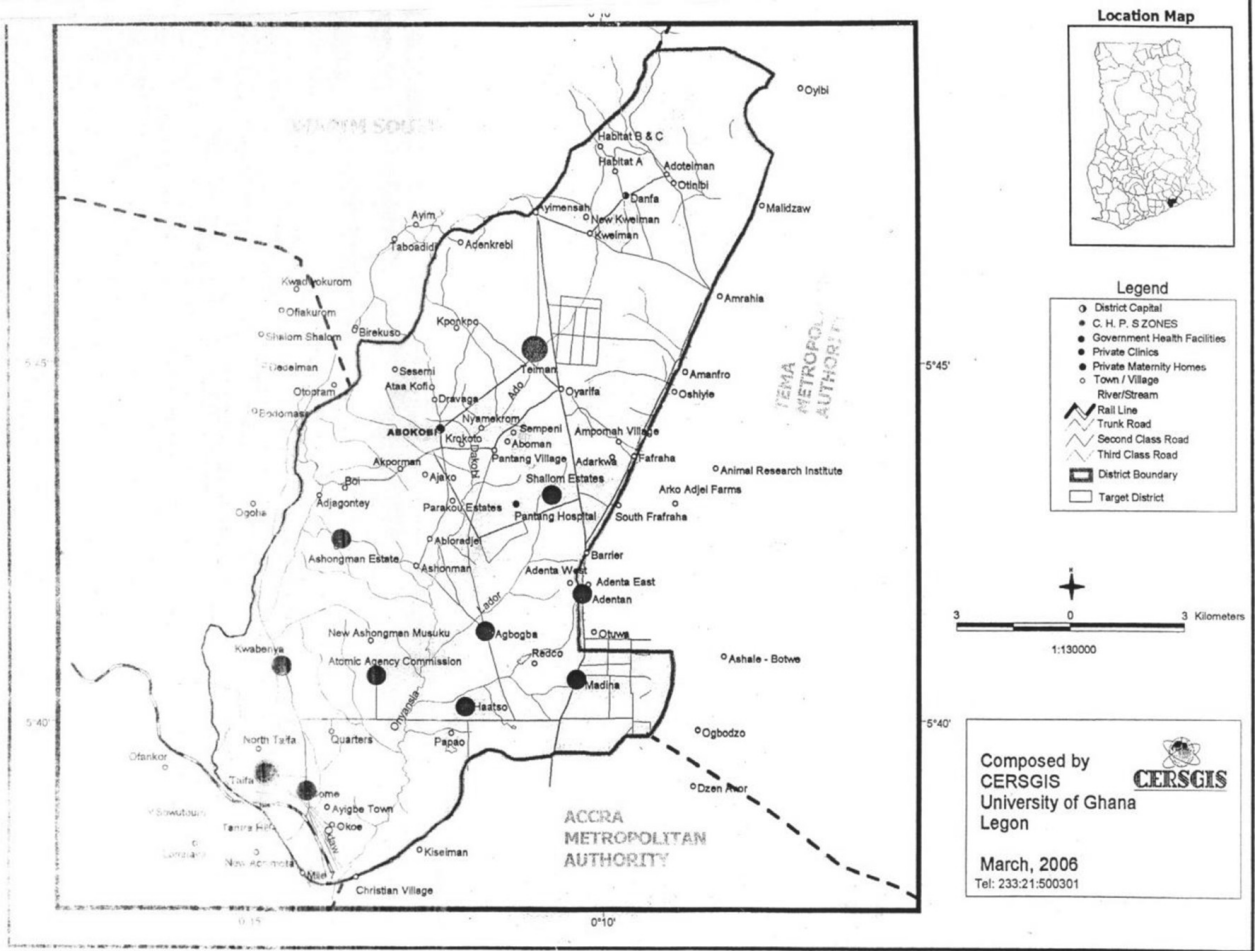
Map showing Ga East Municipal Area.

The Ga East municipality is composed of four sub-municipalities, namely, Madina, Danfa, Taifa, and Dome. It is bounded in the North by Akuapin’s south district, in the West by Ga West, in the East by the Tema municipality, and in the South by Accra Metropolitan Area. It lies in the North-eastern part of the Greater Accra region. The study was conducted in Madina, which is one of the sub-municipalities of the Ga East municipality. Madina is one of the four zonal councils of the Assembly, which is made up of three electoral areas (Nkwantanaa, Tatanaa, and Taatso) having a total population of 108,825. This study concentrated on the Nkwantanaa community of Madina, which has an estimated population of 48,200. It is a mixed settlement comprised of high, medium and low-density residential areas.A total of 39 health facilities are located in the district. Of these, only 6 are public facilities, 31 are private facilities, one is operated by the Christian Health Association of Ghana (CHAG) and one is a quasi-governmental health facility. Public services and trading are the dominant occupations in the municipality, followed by farming and crafts. A sizeable proportion of the workforce in the district is unemployed, which reflects the high poverty level of the area, and makes many people unable to pay for health services that are available (Figure 1).

### Design

The study was descriptive in nature and used mixed methods (quantitative and qualitative) for data collection. The quantitative part of the study consisted of a survey using a questionnaire and the qualitative part used in-depth interviews with key informants. The study used a cross-sectional design that combined systematic and purposive sampling techniques in selecting the study area and the respondents. The Madina sub-municipality was purposely selected because it the most urbanized part of the municipality. The Nkwantanaa electoral area was randomly selected as the study site from among three electoral areas in the sub-municipality. The first household to be surveyed was selected by spinning a bottle, and every fifth house after that was interviewed. Face-to-face interviews were conducted by trained field staff who administered the questionnaires. The questionnaire had four main sections that collected information on: (1) household and demographic characteristics; (2) domestic waste disposal practices; (3) social and cultural perceptions associated with waste disposal; and (4) private sector involvement in waste management. Sections of the questionnaire, particularly the household and demographic characteristics were adopted from the standard DHS questionnaire whiles the others sections were developed by the authors based on the objectives of the study and review of available literature. The questionnaire was pre-tested in the Ga South Municipality that shares similar characteristics with the study district to assessment the suitability of the questions. After the pre-test, some changes were made to the a few questions to make them clearer and more understable.

As mentioned above, the estimated population of the Nkwantanaa electoral area is 48,840. Based on the assumption that 50% of households would dispose of their waste correctly, it was estimated that a sample size of 384 was needed to have a 95% confidence interval with a 5% margin of error. The sample size was rounded to 400 households to make room for the high non-response rate in urban communities. A total of 364 individuals responded to the survey questionnaire, for a non-response rate of 4.9%.

The in-depth interviews were conducted with six key informants who were selected purposively based on their knowledge about the community as it relates to waste management. The people interviewed included four officers from the zonal council of the Madina municipality, one officer from the Department of Environmental Health and Sanitation unit, one Assembly Member, and one officer of a private refuse contractor. The key issues that were discussed included domestic waste disposal practices, private sector involvement in waste management, social and economic factors affecting waste management, and the consequence of poor waste management.

### Data analysis

The data were processed using Epi info software, version 3.4.1, and they were analysed using SPSS, version 16 (IBM, Chicago, IL, USA). Descriptive statistics, such as means, medians, and minimum and maximum values were calculated for continuous variables and percentages were calculated for categorical variables.

The in-depth interviews were audiotaped using a digital audio-recorder, which were complemented with written interview notes. The interviews were subjected to thematic framework analysis, which allows researchers to identify similarities and differences in the qualitative data, before focusing on relationships between different parts of the data [22]. We developed a codebook to group information according to main themes and sub-themes. Based on the codebook, coding of the data were carried out using QSR Nvivo 10^©^, (QSR International, Burlington MA, USA) a computer programme for analysing qualitative data sets. Qualitative trend analysis of the interviews for each topic was used to identify the major issues for each of the main themes and sub-themes. Descriptive narratives supported by illustrative quotes are used to present the results.

## Results

A total of 364 out of the 400 sampled households responded to the survey questionnaire. The sex of the respondents was almost evenly divided, with 49.7% being male and 50.3% being female (see Table 1). The modal age of the respondents was 31-40 years with 40.4% of respondents being in this age group. Nearly half (49.1%) of the respondents had basic education, and 38.2% attained a senior/higher educational level, whereas the rest (12.6%) had no formal education. About three-quarters (73.4%) were employed whilst the remaining 26.6% were unemployed.

**Table 1:** Socio-demographic characteristics of respondents

**Variables**	**Frequency (N = 364)**	**Percent %**
**Sex**		
Male	181	49.7
Female	183	50.3
**Age in years**		
21 – 30	77	21.1
31 – 40	147	40.4
41 – 50	80	22.0
51 – 60	39	10.7
61 and above	21	5.8
**Marital status**		
Single	154	42.3
Married	210	57.7
**Religion**		
Christian	175	48.1
Muslim	155	42.6
Traditional	34	9.3
**Level of education**		
None	46	12.6
Basic education	179	49.1
Senior/Higher	139	38.2

Table 2 shows the characteristics and possessions of the 364 households surveyed in Madina. Most of the respondents (48.1%) received monthly earnings ranging from 100 Ghana Cedis (GH¢100) (US$67) to GH¢399 (US$266), while 21.7% of the respondents received less than GH¢100 (US$67). Roughly 4 out of 10 respondents (39.6%) live in compound houses, a common type of housing in Ghana.

**Table 2:** Household characteristics and possessions of respondent

**Variable**	**Frequency (N = 364)**	**Percent**
**Monthly earnings in GH¢ ($)^**		
Less than 100 (67)	79	21.7
100 – 399 (67-266)	175	48.1
400 – 799 (267-532)	81	22.3
800 above (533 above)	29	8.0
**Residential unit**		
Detached house	90	24.7
Semi – detached house	87	23.9
Flats	43	11.8
Compound house	144	39.6
**Electricity in house**		
Have electricity	269	73.9
Do not have electricity	95	26.1
**People living in your household**		
1 – 4	116	31.9
5 – 9	182	50.0
10 – 14	50	13.7
15 – 19	8	2.2
>19	8	2.2
**Cook at home**		
I cook at home	270	74.2
I don’t cook at home	94	25.8
Cooking schedule*		
Daily	210	77.8
Every other day	32	11.9
Three times a week	15	5.5
Weekly	13	4.8

*Calculated from those who cook at home.

^Cedi/US Dollar exchange rate on interbank exchange rate 2010.

The average household size was 7 people. Half (50.0%) of the respondents surveyed indicated that they had 5 to 9 people living in the same house, whereas 31.9% reported having 1 to 4 people living in the same house. The minimum household size was 3, while the maximum household size was 19. Out of the 364 respondents, 74.2% reported they cooked in the house as opposed to 25.8% who did not cook at home. Not only was the number of respondents who cooked at home high, but the frequency of cooking at home was high. Overall three-quarters (77.8%) of the respondents indicated that they cooked at home daily, 11.9% cooked at home every other day, 5.5% cooked at home three times a week, and 4.8% cooked at home weekly. Only 26.1% of study participants said they did not have electricity in their houses.

Tables 3 and 4 show the types of solid waste generated and the disposal methods used by the households. Food debris was the major waste generated in the study area, with 93.1% of respondents saying they generated food debris as a solid waste. The remaining reported types of waste were: plastics (64.3%), bottles/cans (47.3%), paper (36.0%), and old clothes (21.2%). Most households (82.7%) did not separate their solid waste into different types before disposal, whereas 75% did not cover their waste during storage.

**Table 3:** Types of waste generated by households*

Types of waste generated by household*	**Frequency**	**Percent**
**Food debris**		
	339	93.1
**Plastics**	234	64.3
**Bottles/cans**	131	36.0
**Clothing materials**	78	21.2

*Percentages calculated are from open and multiple responses.

**Table 4:** Waste disposal methods by households

**Characteristics**	**Frequency**	**Percent**
**Separation of solid waste**		
I do not separate my waste	301	82.7
I separate my waste	63	17.3
**Sites of solid waste disposal**		
Appropriate disposal sites	222	61
In – appropriate disposal sites	142	39
**Transportation of waste**		
Self	85	23.4
Children	89	24.5
Housemaid	51	14.0
Paid collection	125	34.3
Others	14	3.8

Similar views were shared during the in-depth interviews.

“If people were willing to sort out from their houses it would make it a bit easier because the contractors have been complaining a lot, and those who put the refuse in the truck also complain about faecal waste being part of the refuse (a male respondent)”.

“The issues of sorting, um.., it is hard to explain because most of the time when we make our rounds we encourage them to do so, but they will always complain that they don’t have time for that and in fact the dust bins are inadequate for such a purpose (a female respondent)”.

Out of the 364 respondents, 61.0% disposed of their waste at the appropriate designated sites, which included the big communal bins and the dump trucks of the paid collection services, while 39.0% of the respondents practiced indiscriminate (crude) dumping (on the streets, in a bush, nearby gutters, or in a hole).

A similar response was confirmed by these quotes:

“The aspect of the crude dumping is very serious, sometimes we find solid waste in gutters, on the street, but we are trying to do something about that” (a male respondent).

“The assembly has prioritized solid waste; formerly people were dumping their solid waste anywhere around the house and even in the gutter and others takes it to the communal dust bin in the market” (a female respondent).

The two most common storage items for solid waste were plastic bins (29.9%) and baskets (25.0%). The remaining respondents reported making use of polythene bags (17.6%), paper boxes (9.1%), old buckets (4.1%), and other items (13.5%). Contracted agents mainly convey household solid waste to the community disposal centre, and 34.3% of respondents said they paid for the collection and transport of their waste. Others transported the waste themselves (23.4%), had their children (24.5%) or housemaids (14.0%) transport it, or used some other means (3.8%). Some of the key informants reported similar information:

“But now the assembly has invested some money and they informed the zonal community and encourage them to register their houses with the private contractors who provide them with twice a week service to collect their solid waste (a female respondent)”.

“Now we have about two active private contractors, they are Amanieh and Zoom Lion, and they are responsible for collection of solid waste from households and transportation to the final disposal site” (a male respondent).

All the 125 of the households who disposed of waste through private contractors indicated that they paid for collection and disposal. This was confirmed by some of the key informants.

*“The private operators charge GH¢8.00 (US$5.36) and GH¢10.00 (US$6.7) respectively” (a male respondent)*.

It was, however, discovered that the charges are made according to type of residential area, by another key informant.

*“In the assembly’s regulations, the private operators are supposed to charge GH¢5 (US$3.35) for 3*^*rd*^*class residential area, GH¢8 (US$5.36) for 2*^*nd*^*class, and GH¢12 (US$8.04) for 1*^*st*^*residential areas a month. It however sometime varies because the contractors normally make special agreements with the households and maybe instead of twice a week collection, they do daily collection which attracts additional fees” (a male respondent).*

“The amount is collected through assembly-managed representatives who collect between 20-50 pesewas, depending on the volume of waste the individual carries, however, I emphasize that it is unofficial (a female respondent)”.

The extent of satisfaction with solid waste management services in the community was low; only 37.1% were satisfied with the services provided. The in-depth interviews also confirmed this finding:

“Even Zoom Lion, they are not meeting the expectations of the people because they are expensive and not everyone can afford it. My little advice is that every assembly has the power to decide whether to work with Zoom Lion or not. Instead it is Zoom Lion that decides what to do in this community (a male respondent)”.

“My people always complain that they are not satisfied with the services provided. Normally, they refuse to do collection twice a week, but sometimes they come after two weeks to collect the refuse, so most times in such situations many people depend on the central containers (a male respondent)”.

“Zoom Lion! They are not working to our satisfaction. The assembly actually has to get more contractors because I think the load is too heavy on Zoom Lion (a female respondent)”.

The majority (76.5%) of the respondents were of the view that solid waste management is important. Most (83.8%) also reported that children were responsible to clean the environment, while 0.5% reported that private contractors should be responsible. Most of the respondents (83.2%) reported that improper solid waste management causes disease or illness, and most of the respondents (83.8%) mentioned malaria. Some 53.6% indicated that they educated their households about good waste management practices. From the multiple responses, 55.8% household said they dispose their waste because of cleanliness, as shown in Table 5.

**Table 5:** Perceptions of households toward solid waste management (n = 364)

**Variables**	**Frequency**	**Percent**
**Do you think waste management is important**		
It is important	261	71.7
It is not important	72	19.8
Do not know whether it is important	31	8.5
**Responsibility to clean**		
Children	305	83.8
Community members	35	9.6
District assembly	22	6.0
Private operators	2	0.5
Disease/illness*		
Cause a disease	303	83.2
Do not cause a disease	56	15.4
Do not know if it cause a disease	5	1.4
Kinds of disease/illness*		
Malaria	171	56.4
Diarrhoea	74	24.4
Typhoid	38	12.5
Others	20	6.6
**Do you educate your household**		
Do education	195	53.6
Do not do education	169	46.4
**Motivation to dispose your waste^**		
Cleanliness	203	55.8
Fear of illness	187	51.4
Smell/odour	31	8.5

*Calculated from 303 respondents who answered yes.

^Percentages calculated are from multiple responses.

The extent of motivation for waste management in the area may be summarized in the following assertion by a key informant.

“We call it waste because we no longer need it. It is not good for humans’ bodies and that’s why we throw it away. Imagine if it was not eliminated - it chokes the gutters and by choking the gutters it serves as a breeding ground for mosquitoes that transmit malaria and other disease” (a male respondent).

“A lot of them, although I am not a health officer, but you know when you are living with waste you can never be healthy. So yes to be frank, Madina as you can see, the town is developing and a lot of people are moving here and businesses are booming here. So if we do not rise up and begin to address this challenge, and in fact, we fear that one day there will be an epidemic” (a male respondent).

Two hundred and seventy eight respondents (76.4%) said they would be willing to pay more when better waste disposal practices are employed, with the remaining 23.6% indicating unwillingness to pay for such service.

## Discussion

The study indicates that increased domestic and household activities in urban environments are linked to the generation of high volumes of domestic wastes [23]. It is also evident that some of this waste is dumped on the streets, gutters, holes and in nearby bushes. This has the potential of serving as breeding grounds for rodents and insects that could increase the risk of the spread of parasitic and zoonotic diseases [24]. Moreover, food debris disposed off indiscriminately could give rise to choked drains and blocked waterways, which create the possibility of flooding during the wet season [25].

The high level of plastic waste generated by households (64.3%) in this study supports the finding that plastic waste generation is increasing in African cities [10]. This phenomenon of increased plastic waste is likely to have implications for disposal, since plastic is not biodegradable. Most often, waste is burnt in the open air at the final disposal sites. Burning of plastic waste will add to the toxic gaseous emissions in the atmosphere, polluting the air and destroying the ozone layer and its protective properties, thereby increasing the risk of health hazards, including cancers. Apart from that, the large quantity of plastic waste that is generated could create financial and socio-economic losses for governments at large when they try to manage it. It is estimated that over 77.9% of households’ generated plastic waste as a component of their domestic waste. In addition, plastic wastes seem to be part of almost all the waste generated at home. This is consistent with earlier studies that suggested that the increased of use of plastics is due to changes in life style and industrialization in which plastic packages replace other forms of packaging [26,27].

The best practice is to store domestic waste in covered plastic bins. However, only 29.9% of the respondents used covered plastic bins to store their solid waste. The use of covered plastic bins protects the waste from direct exposure to flies, vermin, and scavengers, and they also prevent odour nuisances and unsightliness [25,26,28]. The study also reveals that there has not been a significant change from what existed in Accra during 1900-1940 [8]. The pre-1960s philosophy of disposal practices, which was governed by the thinking “out of sight out of mind”, still exists in our waste disposal attitude today. Unfortunately, indiscriminate open dumping of wastes poses significant threats to public health and the environment if they are not stored, collected and disposed of properly [29]. It also makes a travesty of solid waste regulations and defeats the national environmental sanitation policy of maintaining a clean, safe and pleasant physical environment for human settlements [7]. To ensure adherence to the solid waste policies, district, municipal, and metropolitan assemblies will have to develop and strictly enforce regulations in communities.

Most of the respondents did not separate their waste; out of the 364 households, only 63 (17.3%) separated their waste when storing it, while the remaining 301 (82.7%) did not do any kind of solid waste separation, which is a reflection of what happens in most African cities [14,30]. This situation creates a suitable environment for breeding of disease vectors, such as mosquitoes and cockroaches, and the proliferation of rodents, such as rats and mice, which pose threats to public health [31]. The use of colour coded containers to store different types of solid waste, which has been in practice in developed countries for over four decades, is reported to offer a more cost-effective waste management service, since it improves household waste separation and reduces the amount of waste in landfills [29].

Although 61.0% of households seemed to practice appropriate methods of solid waste disposal (i.e., 42.6% used a community bin and 18.4% used paid contractors), 39.0% of the households disposed of their waste in the street, gutters, bushes or any open hole. The Ghana landfill guideline noted that the current practice of solid waste disposal in the country has been largely by uncontrolled dumping in places, such as abandoned quarry sites, valleys, beaches, and drains. These dumping sites are major threats to human health and the environment [32].

The waste collection service in the city is performed by the private sector under various agreements with the metropolitan assembly, as well as the use communal bins provided by private contractors. However, the services provided by the private sector were reported to be unsatisfactory. Overall, 62.9% of households were not satisfied with the solid waste management services in the community. Most respondents complained of irregular patterns in waste collection and the high cost of contracting with private collectors. The Millennium Development Goals provide a framework for assessing the relevance and importance of private sector participation in solid waste management in our efforts to improve the lives of urban dwellers. The impact of private sector participation in solid waste management on these goals cannot be ignored, particularly with respect to Goal 7, which emphasises ensuring environmental sustainability [33,34]. According to the EPA, solid waste services in most developing countries generally do not satisfy the full demand in urban areas [35].

The perceptions of the respondents towards waste management generally seemed to be fairly low. Although 76.5% reported that waste management is important, 83.8% report that it is the responsibility of children to manage waste and not the authorities. Since these people did not see disposal as an important issue, it is not likely that they will improve their waste disposal practices and management practices. This finding, however, is not consistent with other studies that suggested that general waste management in Ghana is perceived as the responsibility of the Ministry of Local Government and Rural Development, which supervises the decentralized MMDAs [18].

The compound houses were densely populated, which may set the pace for the generation of more waste in the community, so the attitudes of a few about waste disposal could result in the whole compound house practicing similar disposal styles or behaviours. Dense populations and increased consumption have been shown to increase more waste and increase disposal problems [18]. The present study also revealed that 19.8% of household heads did not think waste management was important and 8.5% did not know whether it was or not. This could be because 12.6% of them did not have any formal education. This confirms the growing perception in Ghana that low levels of education contribute to poor waste management practices in the country. Other factors that contribute to this situation are poor attitudes, lack of concern about environmental issues, high levels of poverty and misguided waste disposal practices [19]. In Ghana, however, regulatory authority is mainly vested in the EPA under the auspices of the Ministry of Environment and Science. The MMDAs are responsible for the collection and final disposal of solid waste through their Waste Management Departments and their Environmental Health and Sanitation Departments. Increasing rural-urban migration into the Ga East municipality compounds the problem of waste management, as citizens do not take responsibility for adequate waste disposal and, rather, rely on government to dispose of waste. This, in part, may be due to the poor attitudes of the people and their lack of concern about the environment and public health [5].

About 84% of the respondents were aware that improper waste management leads to sicknesses or diseases. This high level of knowledge on the effects of waste management does not correspond with the observed practices. The household heads who educate the occupants of the home have several reasons for properly disposing of waste, including cleanliness, fear of diseases, and odour. The solid waste generated at home was largely food debris and plastic, which are disposed without separation and stored in uncovered plastic bins. Some of the waste is disposed appropriately at communal sites, while some of it is disposed by the practice of crude dumping in gutters, holes, streets, and bushes. Most respondents said they would be happier if more collecting bins were provided and there was regular collection of solid waste for the disposal sites, and some were willing to pay more if the charges were increased. The majority of the households were aware of the health implication of waste, although some had no basic education. Many perceived that children should be responsible for waste management. Most of the respondents thought that improper waste management could lead to malaria and diarrhoea. Proper waste management can lead to improvement in the quality of the environment while, on the other hand, poor waste management can lead to air pollution and breeding of mosquitos, thus causing disease [5,24].

## Conclusions

The study found that the majority of the solid waste generated at home was largely food debris and plastics, which were mainly stored in uncovered plastic containers and disposed without separation. Although waste was disposed appropriately at communal sites, some community members practiced crude dumping in any available space, including gutters, holes, streets, and bushes. Although, indiscriminate dumping was frequently done, the community expressed interest in controlling waste disposal through the use of bins and regular collection to dump sites. The communities cherished improved waste management practices and were willing to pay for improved services. With a little push, support, and education to improve people’s practices and perceptions regarding waste management, some of the challenges confronting municipalities in the area of waste management can be minimized.

### Study limitations

Although this study fills an important gap in the literature, there are a few limitations that are worth noting. The survey did not obtain the determined sample size, due to the fact that some urban dwellers refused to participate in the survey. Out of the 400 respondents who were selected for the sample, 35 respondents refused to participant in the study. Although there was a non-response rate of 4.9%, the data yielded important descriptive information about waste management practices. The qualitative data were derived from highly technical and influential people in the communities who were purposively selected and, therefore, the findings from the in-depth interviews are not necessarily indicative of the situation in all urban communities. Although, there is no reason to doubt the validity of the findings, they could have been augmented by Focus Group Discussions with community members.

## Competing interests

The authors declare that they have no competing interests.

## Authors’ contributions

RMY and PBA conceived and designed the study; RMY collected the data in the field. RMY and PBA wrote the manuscript. All the authors read and approved the manuscript.

## Pre-publication history

The pre-publication history for this paper can be accessed here:

http://www.biomedcentral.com/1471-2458/14/697/prepub
